# Mercury and Selenium Localization in the Cerebrum, Cerebellum, Liver, and Kidney of a Minamata Disease Case

**DOI:** 10.1267/ahc.20-00009

**Published:** 2020-12-19

**Authors:** Masumi Marumoto, Mineshi Sakamoto, Kohji Marumoto, Shozo Tsuruta, Yoshihiro Komohara

**Affiliations:** 1 National Institute for Minamata Disease, 4058–18, Hama, Minamata, Kumamoto 867–0008, Japan; 2 Department of Dental Material Science, School of Dentistry, Aichi Gakuin University, 1–100 Kusumoto-cho, Chikusa-ku, Nagoya, Aichi 464–8650, Japan; 3 Department of Cell Pathology, Graduate School of Medical Sciences, Faculty of Life Sciences, Kumamoto University, 1–1–1 Honjo, Chuo-ku Kumamoto, Kumamoto 860–8556, Japan

**Keywords:** inorganic mercury, selenium, electron probe microanalysis, localization, Minamata disease

## Abstract

Minamata disease is a methylmercury poisoning caused by consumption of marine food contaminated by man-made methylmercury environmental pollution, and its most prominent feature is marked pathological changes in the central nervous system. Morphological alterations are less pronounced in the liver and the kidney, although their mercury levels are higher than those of the brain. In marine mammals, methylmercury is known to be easily converted to inorganic mercury and it combines with selenium forming mercury selenide, which may counteract the toxicity of mercury. However, little is known about the formation of mercury and selenium complex in human organs. In the present study, we examined the cerebrum, cerebellum, liver, and kidney of a Minamata disease case to study the mercury and selenium localization using electron probe microanalysis. Our results indicated the mercury and selenium localization in the specified tissue of the brain, liver, and kidney such as glial cells, Kupffer cells, and renal tubules.

## I Introduction

Minamata disease was caused by ingestion of fish and shellfish contaminated with high levels of methylmercury (MeHg) [16]. The most prominent feature of Minamata disease is significant pathological changes in the central nervous system [16]. Although mercury levels the liver and kidney are higher than those in the central nervous system, the pathological changes are not as pronounced [16]. The patients show the lesions in the cerebellar cortex, calcarine cortex, precentral gyrus, postcentral gyrus, and transverse temporal gyrus [16]. Histopathologically, a characteristic loss of neurons with gliosis in the cortical II and III layers is seen in the cerebrum regardless the severity. The characteristic cerebellar changes are loss of granular cells, and the relatively well-preserved Purkinje cells [16].

Although the mercury in the organs are predominantly MeHg in the early stages of the MeHg exposure, the proportion of inorganic mercury (I-Hg) increases gradually with time [8, 18]. Therefore, in both the acute and subacute cases, the proportion of MeHg to total mercury (T-Hg) in the body is high, but the proportion becomes low in the chronic cases. Takeuchi and Eto [14] reported that the average T-Hg and MeHg concentrations in the brain of the acute cases were 12.6 and 5.61 ppm, and those of the long-term follow-up cases were 3.16 and 0.24 ppm, respectively.

Ganther *et al.* [5] demonstrated that the selenium (Se) played the protective role against the MeHg toxicity in the studies using marine mammals and experimental animals. The concentrations of T-Hg, I-Hg, MeHg, and Se in the organs of Niigata Minamata disease patients have been reported [3], and a strong correlation between mercury and Se concentrations has been shown in the liver [3].

In clinical human samples, the existing of I-Hg was reported using photoemulsion and autometallography [2, 10]. In Minamata disease cases, the localization of I-Hg was visualized by photoemulsion [15]. However, the histological localization of MeHg in the central nervous system and various organs has not been elucidated, because the method of the MeHg visualization is not developed. In addition, the staining method of Se, which may decrease the MeHg toxicity by forming a chemical complex with I-Hg, has not been reported.

Electron probe microanalysis (EPMA) captures the distribution of metallic elements in tissue sections [6, 17], and has been used to detect the distribution of relatively high concentrations of elements in diseases such as primary biliary cirrhosis [6], Wilson’s disease, and occupation-related lung diseases [13, 17], as well as in the skeletal muscle of dolphins [11]. In this study, we used EPMA to examine the localization of mercury and Se in tissues from a Minamata disease case.

## II Materials and Methods

### Case outline

This patient was a Japanese male in his 30s who died in 1958. He had experienced numbness in the upper limbs for approximately 30 mo prior to his death, but did not receive treatment. Approximately 20 days before death, he developed symptoms characteristic of Minamata disease and was diagnosed clinically. As controls, 3 cases other than Minamata disease, which were dissected in 1958–1960, were used. This study is described in accordance with guidelines on patient information protection in the case report of the Japan Pathological Association, and was approved by the National Minamata Disease Research Center Research Ethics Review Committee.

### Preparation and staining of tissue specimens

Blocks of paraffin-embedded were prepared shortly after the patient’s death. Blocks of cerebral calcarine cortex, cerebellum, liver, and kidney sections were sliced into 3-μm serial sections with a microtome and stained with hematoxylin and eosin. The sections were subjected to autometallography [2].

### EPMA analysis

Serial sections were affixed to a carbon sample stage (Niigata Science, Niigata, Japan), deparaffinized, and dried. The specimens were sputter-coated with carbon prior to elemental analysis. Scanning electron microscopy was performed to assess morphological changes, and energy-dispersive X-ray spectroscopy was conducted to determine the elemental composition using an electron probe microanalyzer (JXA-8530F, JEOL, Tokyo, Japan) with an acceleration voltage of 25 kV. Next, we used EPMA for elemental mapping of mercury and Se with 256 × 256 pixel mapping. The accelerating voltage and beam current were set to 15 kV and 0.6 μA, respectively.

### Measurement of mercury concentrations

Formalin-fixed liver, kidney, cerebrum and cerebellum were used for analysis. T-Hg concentrations were determined by cold vapor atomic absorption spectrophotometry according to a previously described method [1]. MeHg concentrations were measured by the Akagi method [1]. I-Hg was calculated as T-Hg minus MeHg. Se concentrations were measured using an inductively coupled plasma mass spectrometer equipped with a collision cell. T-Hg in standard reference material, DORM-2 (Dogfish Muscle; National Research Council, Ottawa, Canada), was measured as quality control, and the measured results fell within the certified range of 4.64 ± 0.26 μg/g. Dogfish muscle DORM-2 was used as the standard reference material for MeHg determinations, and the measured results fell within the certified range of 4.47 ± 0.32 μg/g. NIST 1577 (Bovine Liver; Gaithersburg, USA) was used as a quality control of Se measurement, and the obtained results fell within the certified range of 0.73 ± 0.06 μg/g.

## III Results

### Histopathology

**Fig. 1.**
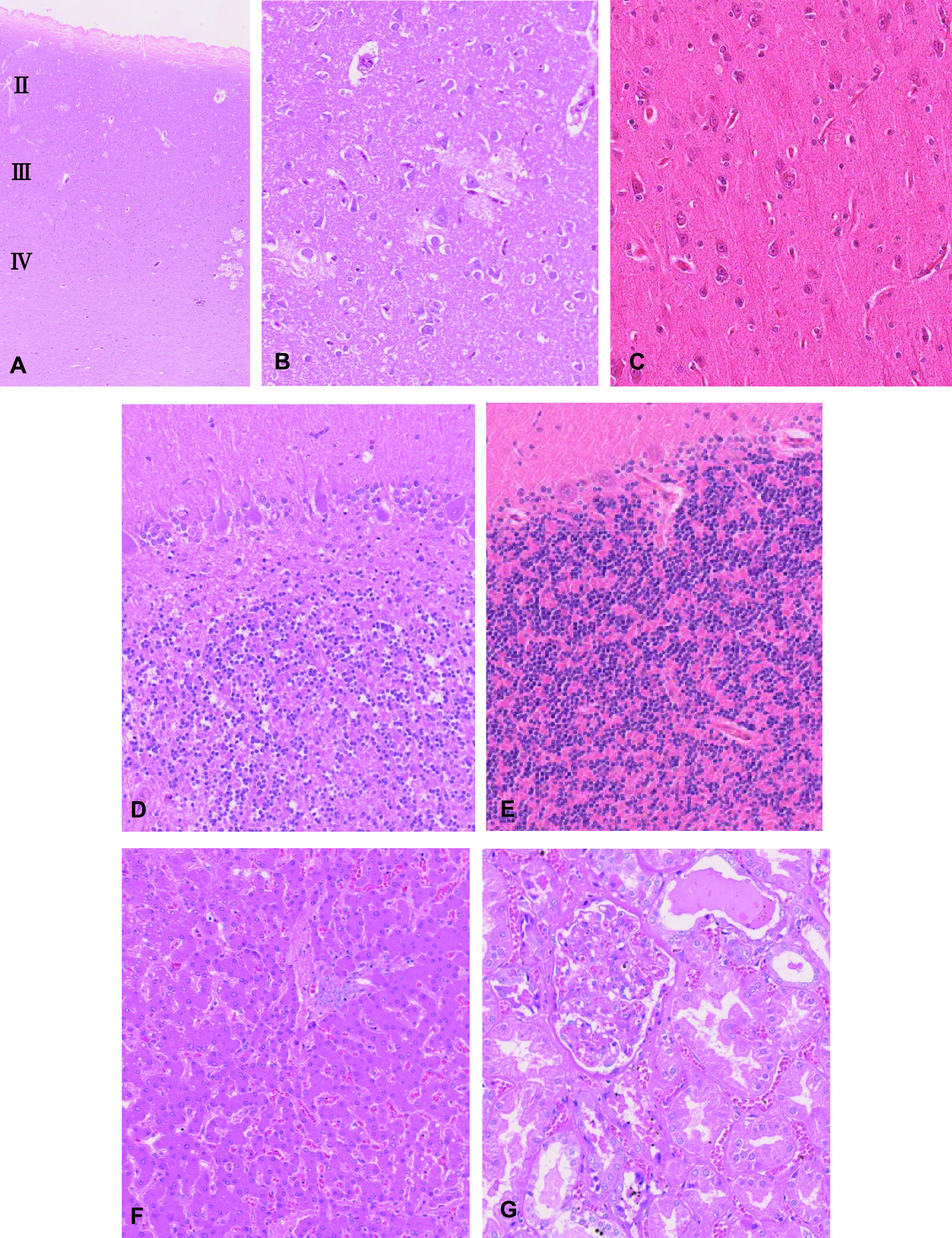
Photomicrographs of calcarine cortex and cerebellum from a Minamata disease patient and control. In the cerebrum, necrosis of cortical layer II to IV neurons and neuropil degeneration (**A, B**). Loss of granule cells beneath the Purkinje cell layer and proliferation of the glial cells were observed in the cerebellum (**D**). In the control case, no significant changes were observed in the cerebrum (**C**) and cerebellum (**E**). There were no significant morphological changes in the liver (**E**) or kidney (**F**) of a Minamata disease patient.

**Fig. 2.**
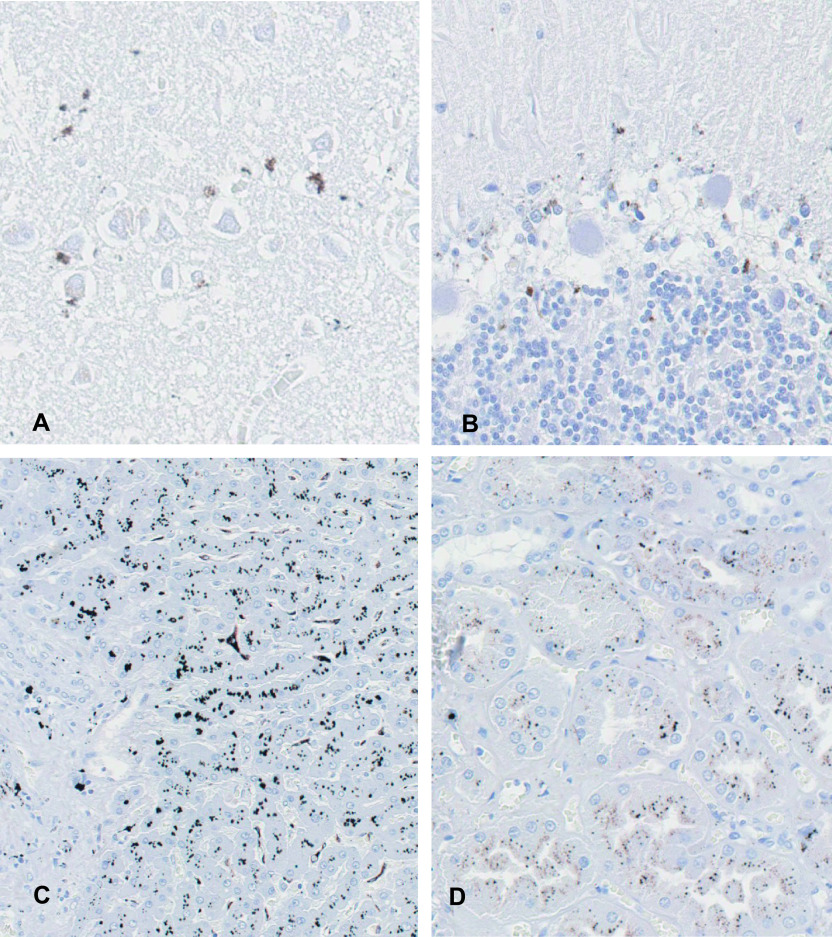
Photomicrographs of autometallography analysis of calcarine cortex, cerebellum, liver, and kidney tissues from a Minamata disease patient. In the cerebrum, multiple inorganic mercury granules were observed in the microglial cells of the calcarine cortex (**A**). In the cerebellum, inorganic mercury was detected in the glial cells (**B**). In the liver, mercury was detected in hepatocytes and Kupffer cells (**C**). In the kidney, mercury was detected in the epithelial cytoplasm of the proximal and distal tubules (**D**).

We observed necrosis of cortical layer II to IV neurons and neuropil degeneration in the cerebrum (Fig. 1A, 1B). In the cerebellum, loss of granule cells beneath the Purkinje cell layer and proliferation of the glial cells were observed (Fig. 1C). There were no significant changes in the liver and kidney (Fig. 1F, 1G). Autometallography detected I-Hg in the microglial cells of the calcarine cortex (Fig. 2A) and the glial cells of the cerebellum (Fig. 2B). Multiple I-Hg granules were observed in the hepatocytes and Kupffer cells (Fig. 2C). In the kidney, I-Hg was detected in the epithelial cytoplasm of the proximal and distal renal tubules (Fig. 2D).

**Fig. 3.**
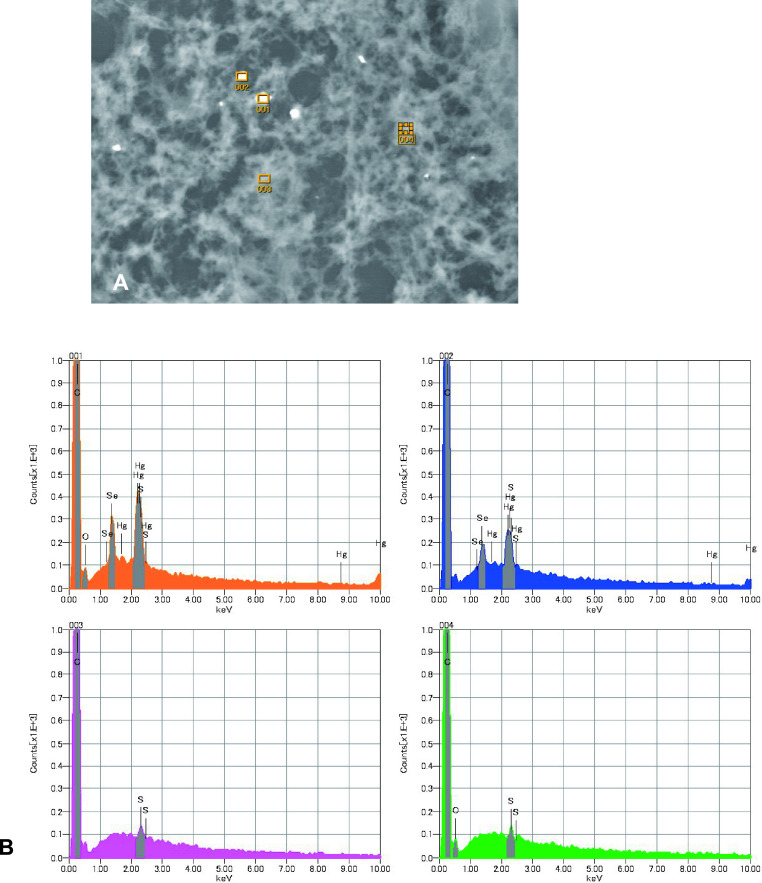
(**A**) Scanning electron microscopy showed white granular materials, indicating metal aggregation in the kidney. (**B**) Energy-dispersive X-ray spectroscopy showed that these white granules contained mercury and selenium (001, 002). Mercury and selenium were not detected in areas lacking white granules (003, 004).

Scanning electron microscopy showed white granular materials that indicated metal aggregation (Fig. 3A) in all organs tested; these white granules contained mercury and Se (Fig. 3B). No evidence of metal aggregation was observed in the organs of control cases.

### Elemental mapping of mercury and Se

Mercury was detected in the glial cells of the calcarine sulcus; some glial cells also contained Se (Fig. 4B, 4C). Neither mercury nor Se were found in the medulla. Mercury and Se were detected in the glial cells in the cerebellum (Fig. 4E, 4F). Mercury and Se were detected in Kupffer cells and hepatocytes; concentrations were higher in Kupffer cells (Fig. 4H, 4I). In the kidney, high concentrations of mercury and Se were found in the proximal and distal tubules, and their distribution was almost identical. None were found in glomeruli or the collecting ducts (Fig. 4K, 4L). Detection of mercury and selenium was confirmed in the organs of Minamata disease cases other than this case (data not shown).

### Mercury and Se concentrations

Table 1 lists mercury and Se concentrations in the organs tested. Relatively high concentrations of mercury were detected in the liver and kidney, mostly in the form of I-Hg. Although the mercury concentration in the brain was lower than in the liver and kidney, approximately 50% of it was MeHg.

## IV Discussion

To our knowledge, this is the first report which indicated the mercury and selenium localization in the specified tissue of the brain, liver, and kidney such as glial cells, Kupffer cells, and renal tubules using an EPMA method.

In this case, lesions characteristic of Minamata disease were observed in the cerebrum and cerebellum, but inorganic mercury and selenium were distributed in diffuse glial cells regardless of the severity of the lesion. In the liver and kidney, mercury and Se were distributed in the same locations, suggesting that I-Hg converted from MeHg makes a mercury and selenium complex which is presumably the mercury selenide (HgSe). However, the formation of HgSe has not been reported in patients of MeHg poisoning.

Selenium has been shown to protect against MeHg toxicity in animal experiments [7, 12]. In dolphin, ingested MeHg binds to various compounds (proteins), and converts to I-Hg and to an insoluble HgSe mineral termed tiemannite [9]. We hypothesized that the formation of HgSe may play a role for Se protection against MeHg poisoning in humans, too.

HgSe has been detected in the brain of several cetacean species, suggesting that MeHg may be demethylated and converted to HgSe in the brain [4, 9]. The HgSe formation may also happen in humans. Our result showed that Se was co-localized in some cerebral microglial cells where mercury deposition was observed. However, only mercury was detected in some microglial cells, suggesting the presence of chemical forms of mercury other than HgSe.

Inorganic Hg has been shown to exert toxic effects on the kidney [19]. Although the kidney concentration of I-Hg in our sample was high (101 ppm), no lesions were observed histopathologicaly, suggesting a protective effect by Se. In this study, high concentrations of mercury and Se were detected in the renal proximal tubules and distal tubules. In cetaceans, the liver is known to demethylate MeHg [7]. Rather than being demethylated in the renal tubular epithelial cells, it is likely that MeHg demethylated to I-Hg in the liver was reabsorbed by the tubular epithelial cells and bound to Se.

The EPMA analysis method developed by Watanabe does not discuss the detection limit [17]. Since the method uses paraffin-embedded sections, it is thought that some degree of metal aggregation is required. Therefore, it is considered that whether or not the presence or absence of local aggregation can be detected is determined rather than the concentration in the entire organ.

We analyzed the samples which were derived from a Minamata disease patient who was autopsied in 1958, and our results indicated the localization of mercury and selenium in the brain, liver and kidney. This study also suggest that even paraffin blocks stored for decades can still be useful to provide new histopathological aspects using a modern techniques of analysis.

## V Conflicts of Interest

The authors declare no potential conflicts of interest with respect to the research, authorship, and publication of this article.

## VI Author Contributions

MM and MS contributed to the study design; MM, KM, and ST acquired, analyzed, and interpreted the data; MM drafted the manuscript; MS, KM, ST, and YK critiqued and revised the manuscript. All authors approved the final draft and agreed to be accountable for all aspects of the work, to ensure that questions relating to the accuracy or integrity of any part of the work would be appropriately investigated and resolved.

## VII Acknowledgments

The authors are grateful to Dr. Yoshifumi Takahashi, Nanae Ryuzaki and Satsuki Nagase, Department of Dental Material Science, Dentistry School of Aichi Gakuin University, Nagoya, Japan. We want to thank Mao Uchikakoi, Miwa Chijiiwa and Ai Motoyama, National Institute for Minamata Disease.

## Figures and Tables

**Fig. 4.**
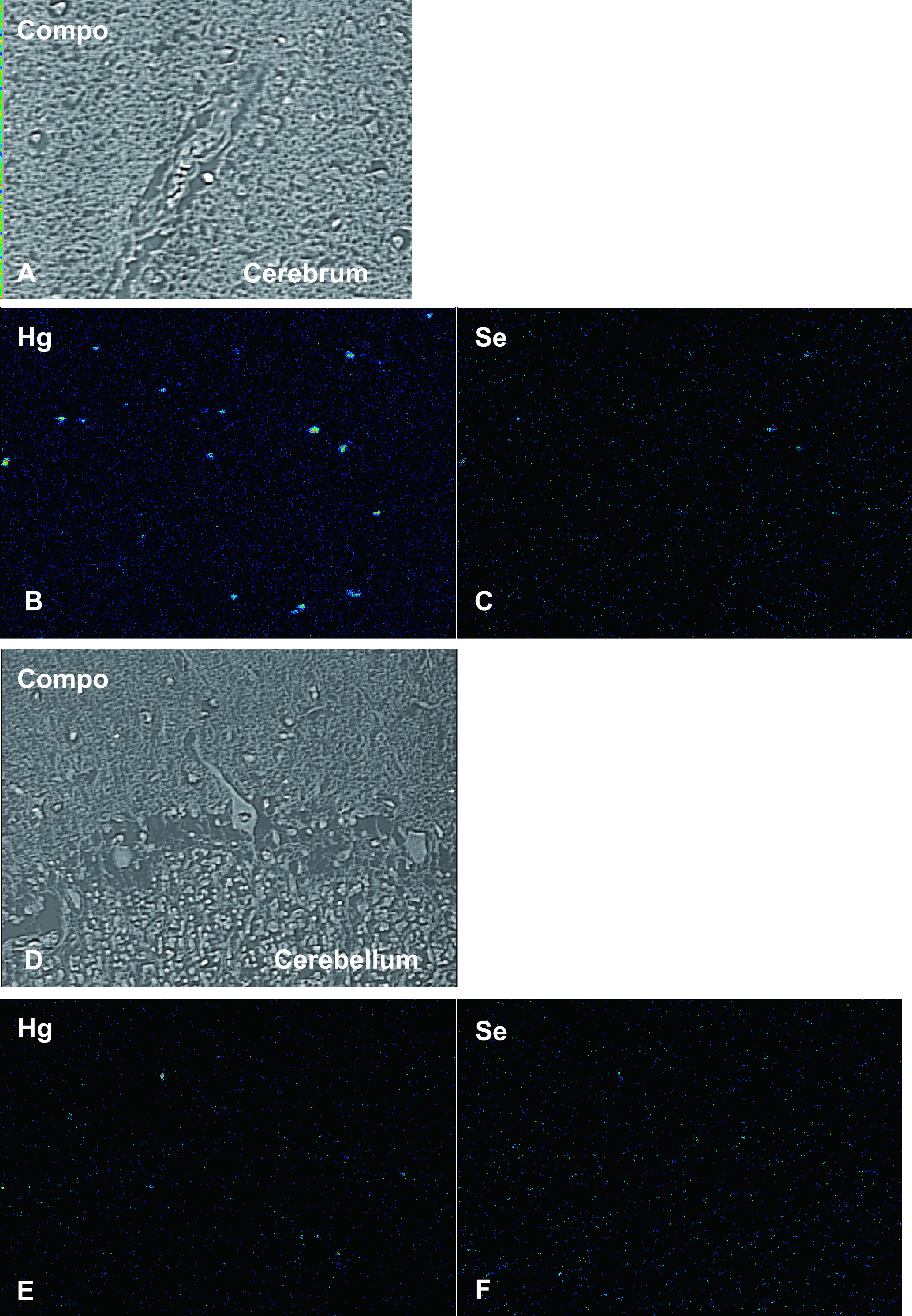
Compositional image in backscattered electron mode (**A, D, G, J**) and electron probe microanalysis mapping of mercury (**B, E, H, K**) and selenium (**C, F, I, L**). (**A–C**) Calcarine cortex. Mercury was detected in the glial cells of the calcarine sulcus; selenium was detected in some glial cells. Neither mercury nor selenium were detected in the medulla. (**D–F**): Cerebellum. Mercury and selenium were detected in the glial cells. (**G–I**): Liver. Mercury and selenium were detected in Kupffer cells and hepatocytes, particularly in Kupffer cells. (**J–L**): Kidney. Mercury and selenium were present in the proximal and distal tubules, and their distribution was almost identical. None were detected in glomeruli or collecting ducts.

**Fig. 4.**
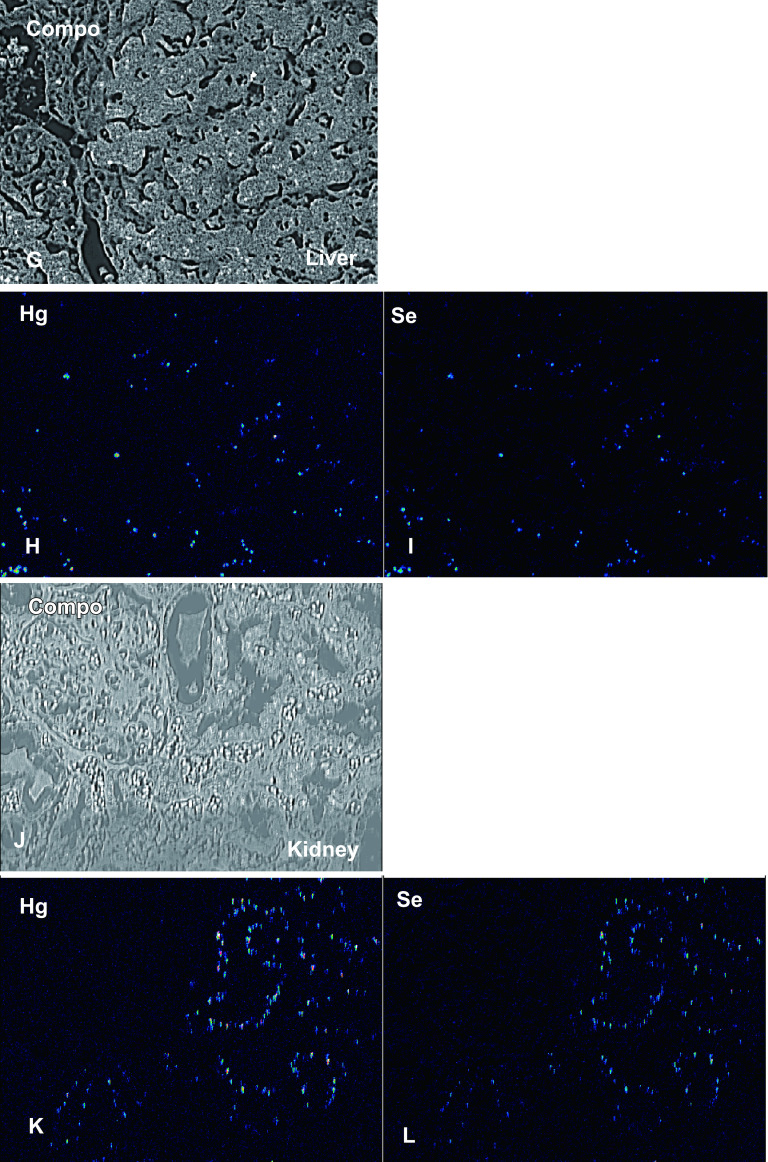
Continued.

**Table 1.** Concentrations of mercury and selenium in tissues from a Minamata disease patient

Tissue	Total mercury	Methylmercury	Inorganic mercury	Selenium
Cerebrum	13.8	5.00	8.80	0.85
Cerebellum	12.8	6.00	6.80	0.98
Liver	46.2	8.45	37.8	20.8
Kidney	111	10.0	101	12.0

Units are microgram per gram of wet weight.

## References

[1] Akagi H., Castillo E. S., Cortes-Maramba N., Francisco-Rivera A. T. and Timbang T. D. (2000) Health assessment for mercury exposure among schoolchildren residing near a gold processing and refining plant in Apokon, Tagum, Davao del Norte, Philippines. Sci. Total Environ. 259; 31–43. 11032133 10.1016/s0048-9697(00)00547-7

[2] Danscher G. and Møller-Madsen B. (1985) Silver amplification of mercury sulfide and selenide: a histochemical method for light and electron microscopic localization of mercury in tissue. J. Histochem. Cytochem. 33; 219–228. 2579122 10.1177/33.3.2579122

[3] Eto K., Takahshi H., Kakita A., Tokunaga H., Yasutake A., Nakano A., et al. (2007) Pathological and biochemical studies of 30 Niigata autopsy cases related to Minamata disease. Jpn. J. Hyg. 62; 70–88. 17334095

[4] Gajdosechova Z., Lawan M. M., Urgast D. S., Raab A., Scheckel K. G., Lombi E., et al. (2016) In vivo formation of natural HgSe nanoparticles in the liver and brain of pilot whales. Sci. Rep. 6; 34361. doi: 10.1038/srep34361. 27678068 10.1038/srep34361 PMC5039623

[5] Ganther H. E., Goudie C., Sunde M. L., Kopecky M. J., Wagner P., Oh S.-H., et al. (1972) Selenium: relation to decreased toxicity of methylmercury added to diets containing tuna. Science 175; 1122–1124. 5062150 10.1126/science.175.4026.1122

[6] Kobayashi M., Watanabe K. and Miyakawa O. (1996) A new sampling technique to obtain the elemental mapping of a bio-tissue section by means of the EPMA equipped with WDX. Niigata Dent. J. 26; 29–37.

[7] Lailson-Brito J., Cruz R., Dorneles P. R., Andrade L., Azevedo Ade F., Fragoso A. B., Vidal L. G., et al. (2012) Mercury-selenium relationships in liver of Guiana dolphin: the possible role of Kupffer cells in the detoxification process by tiemannite formation. PLoS One 7; e42162. 22860072 10.1371/journal.pone.0042162 PMC3409158

[8] Lind B., Friberg L. and Nylander M. (1988) Demethylation of mercury in brain. J. Trace Elem. Exp. Med. 1; 49–56.

[9] Nakazawa E., Ikemoto T., Hokura A., Terada Y., Kunito T., Tanabe S., et al. (2011) The presence of mercury selenide in various tissues of the striped dolphin: evidence from μ-XRF-XRD and XAFS analysis. Metallomics 3; 719–725. 21468440 10.1039/c0mt00106f

[10] Sakai K., Okabe M., Eto K. and Takeuchi T. (1975) Histochemical demonstration of mercury in human tissue cells of Minamata disease by use of autoradiographic procedure. Acta Histochem. Cytochem. 8; 257–264.

[11] Sakamoto M., Itai T., Yasutake A., Iwasaki T., Yasunaga G., Fujise Y., et al. (2015) Mercury speciation and selenium in toothed-whale muscles. Environ. Res. 143; 55–61. 26436307 10.1016/j.envres.2015.09.010

[12] Sakamoto M., Yasutake A., Kakita A., Ryufuku M., Chan H. M., Yamamoto M., et al. (2013) Selenomethionine protects agains neuronal degeneration by methylmercury in the developing rat cerebrum. Environ. Sci. Technol. 47; 2862–2868. 23398308 10.1021/es304226h

[13] Takada T., Moriyama H. and Suzuki E. (2014) Elemental analysis of occupational and environmental lung diseases by electron probe microanalyzer with wavelength dispersive spectrometer. Respir. Invest. 52; 5–13. 10.1016/j.resinv.2013.05.002 24388365

[14] Takeuchi, T. and Eto, K. (1999) Symptomatology and Diagnosis. In “The pathology of Minamata disease—A tragic story of water pollution”, ed. by T. Takeuchi, K. Eto, H. Nakayama and A. Sumiyoshi. Kyusyu University Press, Inc, Fukuoka, Japan, pp. 11–13.

[15] Takeuchi T., Eto K. and Tokunaga H. (1980) Mercury level and histochemical distribution in a human brain with Minamata disease following a long-term clinical course of twenty-six years. Neurotoxicology 10; 651–657. 2562539

[16] Takeuchi T., Morikawa N., Matsumoto H. and Shiraishi Y. (1962) A pathological study of Minamata disease in Japan. Acta Neuropathologica 2; 40–57.

[17] Watanabe K. and Kobayashi M. (2001) How do we analyze the metallic element distribution in tissue section?—New application of element mapping by EPMA—. Journal of the Surface Science Society of Japan 22; 332–336.

[18] World Health Organization. (1990) International Program on Chemical Safety. Environmental Health Criteria 101, Methylmercury. World Health Organization, Geneva.

[19] World Health Organization. (1991) International Program on Chemical Safety. Environmental Health Criteria 118, Inorganic mercury. World Health Organization, Geneva.

